# Radiation-Induced Undifferentiated Pleomorphic Sarcoma in Thyroid: A Rare Occurrence

**DOI:** 10.1007/s12070-024-05122-8

**Published:** 2024-10-24

**Authors:** Deeksha Sharma, Rajjyoti Das, Shiraj Ahmed

**Affiliations:** 1 MCH Head and Neck Oncology, Dr.B.Borooah Cancer Institute, Guwahati, Assam India; 2 Department of Head & Neck Oncology, Dr.B.Borooah Cancer Institute, Guwahati, Assam India; 3 Department of Pathology, Dr.B.Borooah Cancer Institute, Guwahati, Assam India

**Keywords:** Radiation-induced Neoplasm, Sarcoma, Thyroid, Histopathology

## Abstract

We present a rare case of Radiation-induced sarcoma in the thyroid, in a 53-year-old female, seven years after receiving chemoradiation for SCC esophagus. The disease was non resectable due to carotid encasement and was therefore planned for chemotherapy with doxorubicin.

**Supplementary Information:**

The online version contains supplementary material available at 10.1007/s12070-024-05122-8.

## Introduction

Radiation therapy has become a cornerstone of the oncology treatment, with approximately 50% cancer patients receiving it. However, it comes at the price of its own set of adverse effects.

One such rare, long term complication is development of malignant neoplasms. The first case was reported in 1902, establishing the potential of therapeutic radiation to induce malignancy [1].

Sarcomas, are malignant tumours, originating from mesenchymal cells of connective tissues like bone, cartilage, and muscle.

Radiation-Induced Sarcomas (RIS) [2] is a rare entity, reported in fewer than 1% of patients who receive radiation therapy, most commonly involving the bones, with osteosarcoma being the most common histology [3].

So, occurrence of sarcomas in thyroid, either as a primary disease or radiation induced is extremely rare.

We present a case report of an undifferentiated pleomorphic radiation-induced sarcoma thyroid, developing within seven years of radiotherapy.In our literature search we came across only 2 such previously reported cases [2, 4].

## Case Report

A 53 year female, previously treated case of SCC upper esophagus (20 cm), presented with a recently developed neck swelling. She had received Neoadjuvant chemotherapy followed by chemoradiation, which concluded in January 2017.66 Gy was delivered to the primary disease along with involved and elective upper mediastinal nodal stations as well as bilateral supraclavicular stations.Length of the target volume was to allow 5 cm margin superior and inferior to the tumour limits. T-shaped parallel opposing AP-PA radiotherapy portals were planned.The patient also received concurrent chemotherapy with Paclitaxel and carboplatin. Post-therapy endoscopy and scan showed complete resolution of the disease. The patient remained on follow-up.

Seven years later, she developed a right-sided neck swelling since four months, progressively increasing in size. There was no associated pain or compressive symptoms. On examination, the swelling was approximately 5 × 6 cm on the right side of the anterior surface of neck, approximately 4 cm below lower border of the mandible and 1 cm above the clavicle. Medially the swelling extended 1 cm beyond the midline and laterally till the anterior border of the sternocleidomastoid. Overlying skin was normal and the swelling was fixed, not moving on deglutition. On palpation, it was nontender, firm, hard and immobile.

The swelling was thought to be metastatic and was investigated in the surgery department. Upper GI endoscopy, CECT thorax and abdomen did not reveal any abnormality.CECT Neck showed enlarged thyroid lobe with approximately 60 × 49 mm, centrally necrotic irregularly marinated nodular lesion. FNA from the swelling was reported as Poorly differentiated carcinoma, possibly SCC.

She was then referred to the Head and Neck OPD for further management of the considered metastatic thyroid disease.

**Fig. 1:**
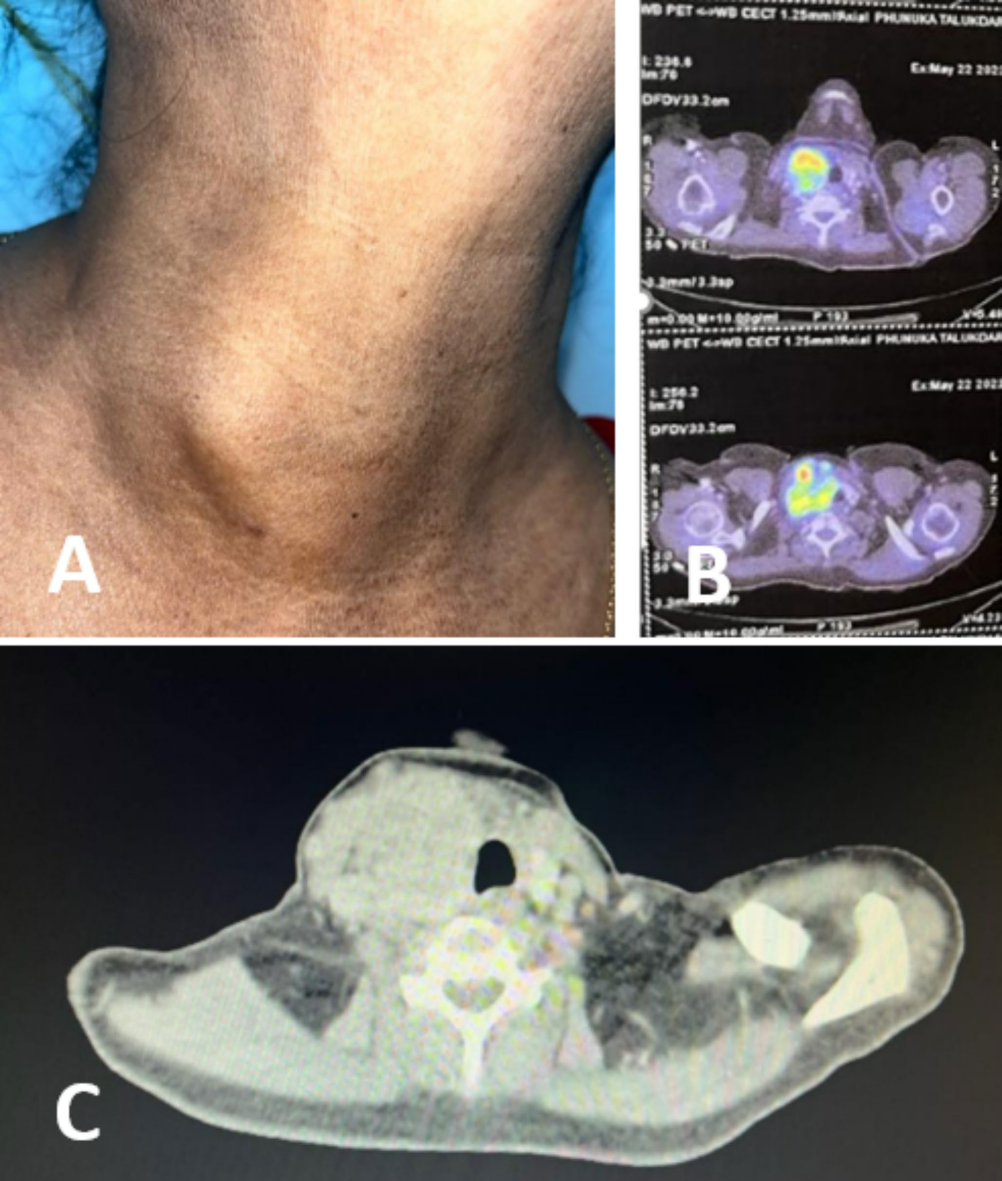
A: Right sided neck swelling B: PET scan showing increased uptake in right suprclavicular region and thyroid gland C: CECT showing centrally necrotic irregularly marinated nodular lesion confluent with right thyroid lobe

PET scan showed, increased FDG uptake in right supraclavicular region (5.5 × 5.0 × 7.0 cm) with SUV max 12.7 with involvement of right thyroid gland. Arch of contact with right common carotid artery was more than 180 degree, and the mass was seen abutting right brachiocephalic artery. No evidence of disease anywhere else in the body [Figure 1].

**Fig. 2:**
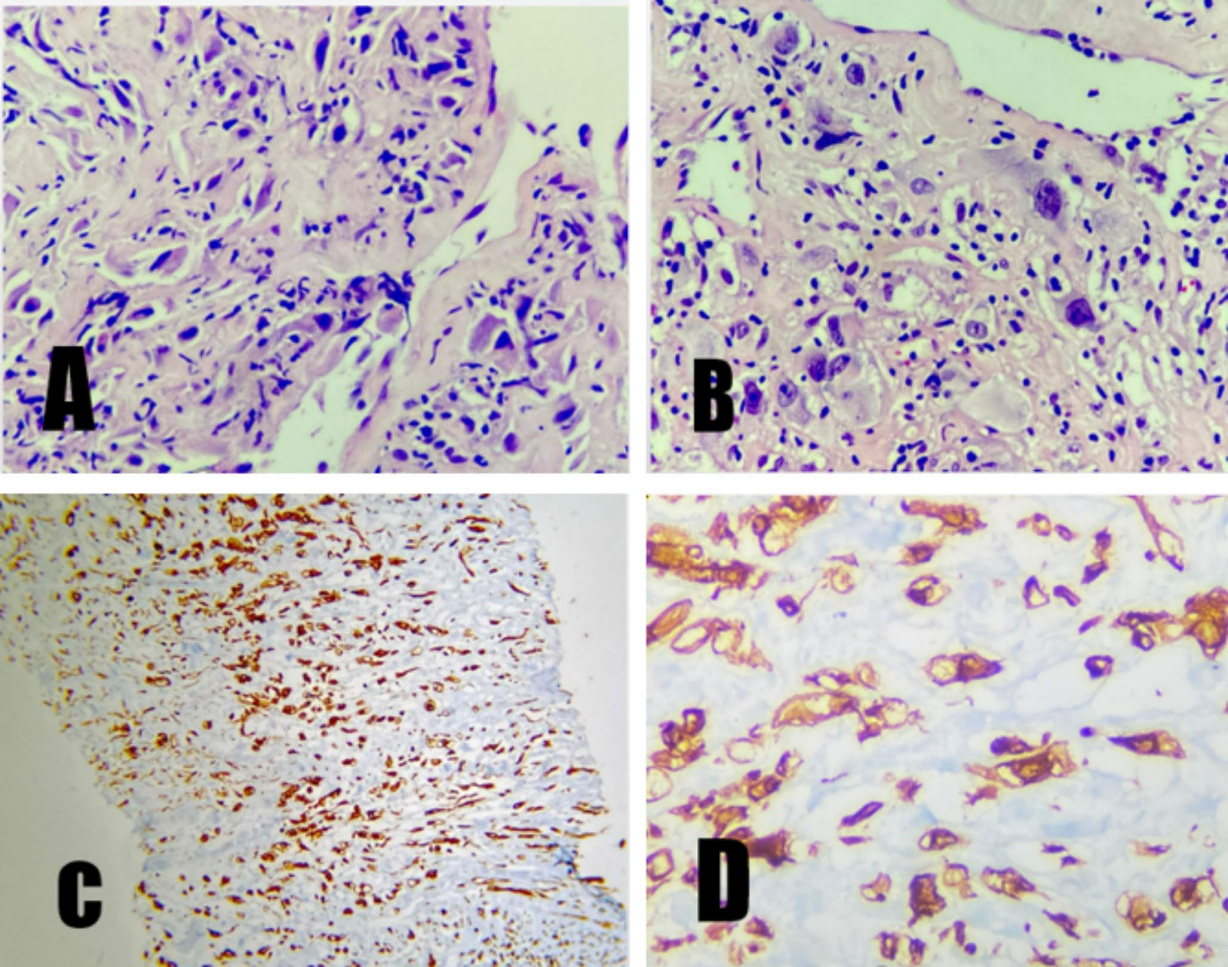
A & B: High power view 40X H & E staining showing atypical spindle cells with hyperchromatic nucleus admixed with many bizarre looking large cells C: IHC positivity (p53 overexpression) for vimentin in spindle cells D: Vimentin positivity in bizarre looking cells

A USG guided core biopsy from the thyroid mass was done with immunohistochemistry markers, that showed vimentin positivity with negative CK, p40, p63,PAX 8.A second panel was applied which was negative for desmin, myogenin, S100, CD34, CD31. P53 was overexpressed and KI 67 was 40% [Figure 2].

After HPE and IHC correlation, final reporting was done as undifferentiated pleomorphic sarcoma, possibly radiation induced.

The case was discussed with a team of multidisciplinary doctors from surgery, radiation and medical oncology department. Due to the inoperability of the disease and unclear role reradiation as the primary modality of treatment, chemotherapy came up as the best possible management option. She is currently planned to receive four cycles of chemotherapy with doxorubicin.

## Discussion

According to the Modified Cahan’s criteria, for a tumour to labelled as Radiation induced malignancy, it must (a) arise in an irradiated field. (b) A sufficient latent period, preferably longer than 4 years, must have elapsed between the initial irradiation and the alleged induced malignancy. (c) The treated tumour and alleged induced tumour must have been biopsied and the two tumours must be of different histology. (d) The tissue in which the alleged induced tumour arose must have been normal (i.e., metabolically and genetically normal) prior to the radiation exposure [5]. 

Radiation is a known risk factor for the development of thyroid cancer. According to the literature, risk is higher during childhood and decreases with increased age at exposure, being low in adults. Papillary carcinoma is the most common histology seen [6]. According to a pooled analysis, the risk is highest at a dose of 2–9 Gy, plateaus at 10–30 Gy and declines at doses greater than that [7].It is believed that higher doses sterilize the thyroid gland, leaving few clonogenic cells able to undergo malignant degeneration. The development of thyroid cancer after high dose radiation can attributed to the theory of accelerated stem cell repopulation compensating for cell killing.

Although there are case reports of thyroid malignancy, mostly papillary after therapeutic radiation, there are only 2 previous reports of thyroid sarcomas developing post-radiation. The first one was reported in 1989, a case of liposarcoma thyroid developing 12 years after receiving high-dose radiotherapy for a T2N1 lymphoepithelioma of the nasopharynx. The second case was that of a low-grade myxofibrosarcoma of the thyroid that developed 27 years after radiotherapy for a localized undifferentiated nasopharyngeal carcinoma (UCNT).

Our case is a case of Undifferentiated pleomorphic sarcoma, probably more aggressive, developing within seven years of receiving chemoradiation for SCC oesophagus.

**We did not find any literature on chemotherapy independently inducing sarcoma development**,** though there are reports of developing the disease when used concurrently with radiation.**

Undifferentiated pleomorphic sarcoma (UPS) is a high grade aggressive soft tissue sarcoma, previously referred as Malignant fibrous histiocytoma (MFH). The 2020 World Health Organization classification incorporated UPS under malignant tumors of uncertain differentiation, and hence its diagnosis is established after exclusion of other soft tissue sarcomas [8]. Histopathological examination is the key to diagnosis with core needle biopsies preferred to provide adequate tissue sample.

On microscopy, UPS exhibits atypical, pleomorphic spindle cells with abundant mitotic figures. The definitive diagnosis is confirmed by excluding other malignancies with a panel of immunohistochemical markers that include keratins, S100 protein, SOX10; smooth muscle actin (SMA); and desmin, expressed in metastatic sarcomatoid carcinoma, melanoma, and pleomorphic myogenic sarcomas, respectively. Vimentin, p53, and Ki67 are other reportedly expressed IHC markers in UPS [9]. 

Our case showed vimentin positivity, p53 overexpression and negativity for other markers. Hence, correlating the histology and excluding other sarcomas, the diagnosis of UPS was established.

There are no standardized guidelines for the management of Radiation-induced sarcomas, thus, making it challenging. The standard treatment for sarcomas is surgical excision followed by adjuvant treatment as needed, making surgery as the first line treatment option.

Among the previously reported cases, one underwent a subtotal thyroidectomy while the other underwent an R1 resection followed by adjuvant RT.

For an unresectable disease, the role of secondary Radiation and chemotherapy in RIS is unclear. Our case was taken up for single agent chemotherapy with doxorubicin with a palliative intent of treatment.

## Conclusion

Owing to the rarity of the occurrence, a clinician does not frequently come across this long term complication of radiation therapy. This report highlights the importance for follow-up at regular intervals post radiation and vigilant examination at each visit. As the saying goes; the eyes don’t see, what the mind doesn’t know’, awareness about this rare entity is needed or there are complete chances of missing some early developing radiation induced malignancy in its early stage. Thyroid being an unusual site for development of sarcomas, a complete and thorough histopathological examination combined with immunohistochemistry is needed for proper diagnosis. Due to very few reports of radiation induced sarcomas in thyroid, there is no standardised management. Hence more evidence is needed regarding non-surgical management in cases of unresectable thyroid sarcomas.

## Electronic Supplementary Material

Below is the link to the electronic supplementary material.

Supplementary Material 1
