# Supplementary material for: Radiation-Induced Undifferentiated Pleomorphic Sarcoma in Thyroid: A Rare Occurrence
Source: Indian J Otolaryngol Head Neck Surg. 2024 Oct 24;77(1):453–7. doi: 10.1007/s12070-024-05122-8 (PMC11890467; doi:10.1007/s12070-024-05122-8)
Supplement: Supplementary file 1 — Supplementary Material 1 [file 12070_2024_5122_MOESM1_ESM.docx]

REVISIONS HAVE BEEN INCORPORATED IN MANUSCRIPT AND HIGHLIGHTED WITH BOLD FONT,

1.RADIATION DETAILS:

**She had received Neoadjuvant chemotherapy followed by chemoradiation, which concluded in January 2017.66 Gy was delivered to the primary disease along with involved and elective upper mediastinal nodal stations as well as bilateral supraclavicular stations.Length of the target volume was to allow 5cm margin superior and inferior to the tumour limits . T-shaped parallel opposing AP-PA radiotherapy portals were planned.The patient also received concurrent chemotherapy with Paclitaxel and carboplatin.**


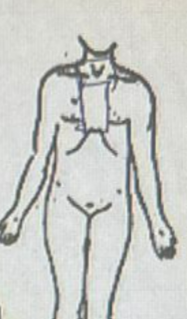
 THE RADIATION FIELD

2. **We did not find any literature on chemotherapy independently inducing sarcoma development, though there are reports of developing the disease when used concurrently with radiation.**
